# India’s Decision to Deny an Extension of Patent for Bedaquiline: A Public Health Imperative

**DOI:** 10.7759/cureus.49542

**Published:** 2023-11-28

**Authors:** Sankalp Yadav, Gautam Rawal, Madhan Jeyaraman

**Affiliations:** 1 Medicine, Shri Madan Lal Khurana Chest Clinic, Moti Nagar, New Delhi, IND; 2 Respiratory Medical Critical Care, Max Super Speciality Hospital, New Delhi, IND; 3 Orthopaedics, ACS Medical College and Hospital, Dr. MGR Educational and Research Institute, Chennai, IND

**Keywords:** patent denial, prexdr tb, xdr-tb: extensively drug resistant tuberculosis, multi-drug resistant tb -mdr, mtb (mycobacterium tuberculosis), bedaquiline

## Abstract

Tuberculosis (TB) remains a major global public health concern, which has become worse in low- and middle-income nations due to the rise of drug-resistant strains of the disease. Bedaquiline, a groundbreaking anti-TB drug, shows great promise for addressing multidrug-resistant TB (MDR-TB). However, the recent decision by India to reject the application for a patent extension on bedaquiline raises crucial questions regarding access to essential medicines and public health requirements. This article examines the significance of India's rejection of the patent extension for bedaquiline, outlining the global implications of this decision and its impact on intellectual property rights, access to medicines, and the future of drug development. India's stance serves as a model for other countries to prioritize public health in their patent-related decisions, underlining the need for a balanced approach to intellectual property rights, innovation, and affordability in the pharmaceutical sector. In the context of the ongoing battle against drug-resistant TB, India's decision on bedaquiline signifies the evolving landscape of pharmaceutical patent practices in the service of global public health.

## Introduction and background

In endemic countries, tuberculosis (TB) continues to be a major health concern [1]. The emergence of drug-resistant TB strains has exacerbated the situation, necessitating innovative solutions for effective treatment [2]. Bedaquiline, a novel anti-TB drug, has shown promise in tackling multidrug-resistant TB (MDR-TB) [3]. However, a recent decision by India to reject the application for an extension of the patent on bedaquiline raises important questions regarding access to essential medicines and public health needs [4].

Bedaquiline, a diarylquinoline compound, is a groundbreaking drug that specifically targets the mycobacterial Adenosine triphosphate (ATP) synthase, disrupting energy production in *Mycobacterium tuberculosis* [5]. First approved by the U.S. Food and Drug Administration (FDA) in 2012, bedaquiline has offered renewed hope in the battle against MDR-TB, a disease characterized by resistance to two or more of the first-line TB drugs [6]. MDR-TB poses an imminent danger to public health globally [7]. The protracted and complex treatment regimens, often involving costly second-line drugs with considerable side effects, place a substantial burden on both patients and healthcare systems [8]. The World Health Organization (WHO) has recognized the urgency of addressing this crisis and has recommended the inclusion of bedaquiline in MDR-TB treatment regimens [9]. India, a country with a high TB burden, plays a pivotal role in the global fight against TB [10]. In April 2023, India made a significant decision by rejecting the application for a patent extension on bedaquiline, challenging the conventional norms of pharmaceutical patenting [11]. This move is driven by a commitment to prioritize public health interests, enabling the manufacturing of generic forms of bedaquiline [12]. Pharmaceutical patents are crucial for incentivizing innovation and investment in drug research and development [13]. However, they also influence the affordability and availability of medications, especially in contexts with limited resources [14]. India's decision serves as a reminder of the ongoing debate between the defence of rights to intellectual property and the need for equitable access to essential medicines [15]. India's decision to deny a patent extension on bedaquiline is commendable in the context of public health [16]. It signifies a shift towards ensuring affordable access to a critical drug for MDR-TB treatment. This decision also underscores the importance of robust patent laws that strike a balance between encouraging innovation and safeguarding public health interests [17].

## Review

To expand upon the significance of India's decision to deny the patent extension for bedaquiline, we must look into the broader implications for global health, intellectual property rights, and the future of drug development and accessibility. We searched databases from Scopus and PubMed and thoroughly reviewed almost all available scholarly articles from January 2020 until October 19, 2023, about bedaquiline and the denial of its patent extension in India. After scrutiny of articles, only four articles were found published in indexed journals which were considered for this review. 

Global burden of TB

TB remains a grave public health issue worldwide [18]. The World Health Organization (WHO) estimated that 10 million humans acquired TB in 2020, contributing to 1.4 million fatalities. [1]. Although tremendous strides have been made in the fight against the illness, it continues to disproportionately affect low and middle-income countries [18]. A significant challenge in the fight against TB is the growing number of strains that are resistant to drugs. Resistant to no less than isoniazid and rifampicin, which are two of the most efficacious first-line TB medications, is what is known as MDR-TB. Extensively drug-resistant TB (XDR-TB) is an even more severe form, with additional resistance to second-line drugs. These drug-resistant forms of TB are more challenging to treat and can lead to higher mortality rates [2].

Challenges in MDR-TB treatment

Managing MDR-TB is a complex undertaking that poses substantial challenges. Traditional TB treatment regimens last six to nine months and rely on first-line drugs, which are relatively affordable. However, MDR-TB treatment regimens are prolonged, lasting from 12 to 24 months, and require a mix of first-line and second-line medications. The complexity and duration of MDR-TB treatment significantly increase the risk of poor patient adherence, as long-term medication can be burdensome, and the side effects of second-line drugs can be severe [3].

The role of bedaquiline

Bedaquiline, introduced in 2012, marked a significant milestone in TB treatment [4]. It is the first new TB drug to be approved in decades and is specifically designed to combat MDR-TB. By inhibiting mycobacterial ATP synthase, it targets a unique aspect of the TB bacterium's metabolism, making it highly effective against drug-resistant strains [5]. The usual dose of bedaquiline is 400 mg daily for two weeks followed by 200 mg alternate day for 22 weeks. In clinical trials, bedaquiline demonstrated a significant increase in treatment success rates for MDR-TB patients [6].

Global response to drug-resistant TB

Recognizing the gravity of drug-resistant TB, the WHO declared it a public health emergency in 1993 [7]. Since then, international efforts have focused on improving diagnostic tools, developing new drugs, and formulating treatment guidelines [8]. The global response includes the End TB Strategy and the Global Plan to End TB 2016-2020, which emphasizes the importance of addressing MDR-TB [9].

The benefits of having access to essential drugs

One of the key elements in controlling TB, particularly drug-resistant TB, is ensuring access to essential medicines. This access is hindered by various factors, including the high cost of drugs, limited availability of diagnostic tools, and intellectual property rights. The issue of access to medicines raises a fundamental question: how can we balance the need for innovation and the defense of intellectual property with the imperative of providing affordable and equitable access to life-saving treatments? [15].

Pharmaceutical patents and their impact on access

Pharmaceutical patents play a dual role in the world of medicine. On one hand, they incentivize innovation by providing a period of market exclusivity, during which drug developers can recoup their research and development investments. On the other hand, patents can hinder access to essential medicines, as they grant the patent holder the exclusive right to produce and sell the drug, often at premium prices [13].

The Doha declaration on Trade-Related Aspects of Intellectual Property Rights (TRIPS) and public health

The World Trade Organization (WTO) recognizes the significance of public health in trade agreements and intellectual property rights. The Doha Declaration on the Agreement on TRIPS and Public Health, adopted in 2001, emphasized the authority of nations to implement policies aimed at safeguarding public health and advancing universal access to medications [15]. It reaffirmed the adaptability of TRIPS to accommodate these public health interests [15,19,20].

India's role in access to medicines

Often called the "pharmacy of the developing world," India has played a crucial role in ensuring access to affordable generic medicines, particularly in the treatment of HIV [20]. This reputation is the result of India's legal framework, which grants patents but also includes provisions to safeguard public health interests.

The Indian Patent Act

India's Patent Act, amended in 2005, aligns with the country's obligations under TRIPS [21]. This act provides for the granting of patents on pharmaceuticals and other inventions while also including provisions for compulsory licensing. These provisions allow generic manufacturers to produce patented medicines without the patent holder's approval in situations where access to essential medicines is deemed vital for public health [9].

Bedaquiline's patent history

Bedaquiline's patent history exemplifies the complexities of patent protection and access to medicines. Initially held by Janssen Pharmaceuticals, a subsidiary of Johnson & Johnson, the patents for bedaquiline raised concerns about affordability and access [7]. To address these concerns, Janssen adopted a differential pricing strategy, offering reduced prices in certain countries [7,22]. However, this strategy did not extend to all countries in need [7]. About five patents are involved in relation to bedaquiline (Table 1) [14].

**Table 1:** Five patents are involved in bedaquiline TB: Tuberculosis. Reference [14].

Patent	Purpose
Patent 1	For making the base bedaquiline compound
Patent 2	For using bedaquiline for drug-resistant TB
Patent 3	For using bedaquiline to treat latent TB
Patent 4	For preparation process
Patent 5	Is about the end formulation sold in markets

The patent application for bedaquiline in India

Janssen Pharmaceuticals submitted a patent extension application for bedaquiline in India, seeking to prolong its intellectual property protection. Such an extension would have delayed the entry of generic forms of the drug into the Indian market, potentially limiting affordability and accessibility [7].

India's decision to reject the patent extension

In a historic decision in April 2023, India's Intellectual Property Appellate Board (IPAB) rejected the application for a patent extension on bedaquiline. This decision aligns with India's commitment to public health and has far-reaching implications for the availability of affordable bedaquiline in India and beyond [7].

Key factors influencing India's decision

Several significant factors contributed to India's rejection of the patent extension for bedaquiline:

Commitment to Public Health

India has a long history of prioritizing public health in its policy decisions. The rejection of the patent extension aligns with India's commitment to providing affordable access to essential medicines, particularly for diseases like TB [11].

The TRIPS Agreement

The Agreement on TRIPS allows countries to take measures to protect public health. India's decision to reject the patent extension is consistent with its obligations under TRIPS [15].

Compulsory Licensing

India's Patent Act includes provisions for compulsory licensing, which can be invoked in cases where access to a patented medicine is deemed crucial for public health. While India did not use compulsory licensing in this case, the presence of such provisions may have influenced the decision to reject the patent extension [15].

Price and Access Concerns

The high cost of patented drugs can significantly limit access, particularly in low and middle-income countries. By rejecting the patent extension, India is paving the way for the manufacturing of affordable generic forms of bedaquiline, which can enhance access to this life-saving drug [14].

Precedent for Public Health

India's decision sets a precedent for other countries to prioritize public health in their patent-related decisions, particularly for drugs used in the treatment of infectious diseases [16].

Global implications of India's decision

India's decision to deny the patent extension for bedaquiline has significant global implications:

Improved Access to Bedaquiline

The rejection of the patent extension will likely lead to the availability of more affordable generic versions of bedaquiline in India and other countries that rely on Indian pharmaceutical production [16].

Reduced Treatment Costs

The availability of generic bedaquiline can lower the cost of MDR-TB treatment, reducing the economic burden on healthcare systems and patients [16].

Encouragement for Other Countries

India's decision serves as an example for other countries to prioritize public health when considering patent extensions for essential medicines [16].

Impact on Other Diseases

The decision sets a precedent for addressing access to other essential medicines, particularly those used in the treatment of infectious diseases [16].

The importance of intellectual property rights in drug development

Intellectual property rights, including patents, play a crucial role in incentivizing innovation and investment in drug research and development. Patents grant inventors the sole power to produce, sell, and distribute their inventions for a specific period, typically twenty years from the date of filing. During this time, inventors can recoup their investments and earn a profit [13].

The patent system's influence on access to medicines

While patents incentivize innovation, they can also create barriers to access. Patented medicines are often priced significantly higher than their generic counterparts, limiting affordability, particularly in resource-limited settings. This pricing disparity can affect the ability of individuals and healthcare systems to access essential treatments [13].

The dilemma of access versus innovation

The key argument in the discussion of pharmaceutical patents is the conflict between the necessity of providing incentives for drug innovation and the necessity of guaranteeing access to necessary medications. Striking a balance between these two imperatives is challenging but essential for global health [15].

TRIPS flexibilities and public health

The TRIPS agreement permits flexibilities that allow countries to protect public health while upholding their obligations to intellectual property rights [15]. Some of the key TRIPS flexibilities include:

Compulsory Licensing

TRIPS permits countries to issue compulsory licenses, permitting generic manufacturers to produce patented medicines without the permission of the patent holder. Countries can use this provision when they face public health crises or when they are unable to obtain affordable access to essential medicines [15].

Parallel Importation

Parallel importation permits countries to import patented medicines from other countries where they are available at lower prices [15]. This can enhance access to medicines without the permission of the patent holder [15].

Bolar Exception

The Bolar exception allows countries to permit the production of generic medicines to prepare for market entry as soon as the patent expires [15]. This facilitates faster access to generic forms of drugs [15].

Research Exemptions

TRIPS allows countries to permit the production of patented medicines for research purposes without the permission of the patent holder [15].

The Doha Declaration and TRIPS flexibilities

The Doha Declaration on TRIPS and Public Health, adopted in 2001, clarified that TRIPS should not hinder countries' ability to protect public health. It affirmed the flexibility of TRIPS to accommodate measures for public health, such as compulsory licensing and the production of generic medicines. The Declaration stated that countries have the right to decipher and apply TRIPS in a manner that is consistent with their public health objectives. It reinforced the principle that the availability of medicines is a basic component of the right to health [15].

India's patent system and public health

India's approach to intellectual property rights, particularly in the pharmaceutical sector, has been shaped by its commitment to public health. India has a legal framework that grants patents but also includes provisions to safeguard public health interests [9].

The Indian Patent Act

India's Patent Act, amended in 2005, provides for the granting of patents on pharmaceuticals and other inventions [9]. The Act aligns with the country's obligations under TRIPS [18].

Compulsory Licensing Provisions

India's Patent Act includes provisions for compulsory licensing, allowing generic manufacturers to produce patented medicines in specific situations deemed vital for public health [9].

Balancing Innovation and Access

India's approach to intellectual property rights seeks to balance the need to incentivize innovation and the imperative to ensure access to essential medicines [9].

Access to medicines: a global challenge

Ensuring access to essential medicines is a complex global challenge [23]. Several key factors influence the accessibility and affordability of medicines:

Cost of Drug Development

Developing new drugs is a costly and time-consuming process. Research and development (R&D) expenditure, clinical trials, and regulatory requirements contribute to the high cost of bringing a new drug to commercial market [24].

Patent Systems

Intellectual property systems vary globally and significantly impact the availability and affordability of medicines [25].

Pharmaceutical Industry Practices

Pharmaceutical companies employ various strategies to enhance access to medicines. One common approach is differential pricing, where medicines are offered at lower prices in low and middle-income countries. While this strategy can enhance access, it can also result in price disparities and access barriers [26].

Public Health Imperatives

The imperative to prioritize public health in patent-related decisions is gaining prominence, as demonstrated by India's rejection of the patent extension for bedaquiline [16].

Global Health Disparities

Access to medicines is fundamentally a matter of global health equity and must address disparities between high-income and low and middle-income countries [27].

Potential solutions to enhance access to medicines

Addressing the challenge of access to medicines requires a multifaceted approach:

Government Policies

Governments can implement policies that balance intellectual property protection with public health imperatives. They can use TRIPS flexibilities to release compulsory licenses, import medicines, and ensure that access to essential medicines is not hindered by patents [28].

Research and Development Incentives (R&D)

Governments and international organizations can provide incentives and funding to stimulate research and development in areas with unmet medical needs. This can foster innovation in the development of essential medicines while keeping costs in check [28].

Price Negotiations

Negotiations between pharmaceutical companies, governments, and international organizations can lead to more equitable drug pricing. Such negotiations aim to find a balance between the need to recoup R&D investments and the requirement of ensuring affordable access [29].

Licensing Agreements

Collaborative licensing agreements between pharmaceutical companies and generic manufacturers can expand access to essential medicines while respecting intellectual property rights [29].

Investment in Generic Manufacturing

Developing countries can invest in their generic pharmaceutical industries to promote local production of essential medicines [29]. This can reduce reliance on imported drugs and enhance affordability [29].

Increased Transparency

Transparency in pricing, research and development costs, and licensing agreements can enhance accountability and access. It allows stakeholders to understand the factors influencing drug pricing and access [30].

The global impact of India's decision

India's decision to reject the patent extension for bedaquiline is a watershed moment in the global parley on intellectual property rights and access to essential medicines. Its impact reaches beyond the boundaries of a single drug and country:

A Model for Access-centric Decisions

India's decision can serve as a model for other countries, encouraging them to prioritize public health when considering patent extensions for essential medicines [16].

Increased Access to Bedaquiline

The availability of generic bedaquiline at more affordable prices can improve access to MDR-TB treatment, alleviating the economic burden on healthcare systems and patients [16].

A Precedent for Public Health

The decision sets a precedent for addressing the availability of other essential medicines, particularly those used in the treatment of infectious diseases [16].

A Challenge to the Global Pharmaceutical Industry

The decision poses questions about how the global pharmaceutical industry will adapt to evolving approaches to intellectual property in the context of public health [16].

A Call for Equitable Access

India's decision highlights the importance of balancing innovation incentives with equitable access to essential medicines for global health equity [16].

As drug-resistant TB continues to rise, and with the limitations in the effectiveness and safety of currently employed treatments for MDR-TB, the introduction of bedaquiline marked a significant and innovative contribution to the available array of anti-TB medications [31]. The generic availability of this drug holds particular promise in regions where TB remains highly prevalent. There is the paucity of data on this denial of patent extension and thus it is imperative that large-scale studies aimed at the impact of MDR-TB control, accessibility of bedaquiline, and related aspects be done. The four articles we considered were either editorial, world report, or news [2,4,32,33].

## Conclusions

India's decision to reject the patent extension for bedaquiline marks a pivotal moment in the global dialogue on intellectual property rights, access to medicines, and public health priorities. This decision exemplifies India's commitment to providing affordable access to essential medicines, particularly for diseases that disproportionately affect the country, such as tuberculosis. The rejection of the patent extension has significant implications for access to bedaquiline and serves as a model for other countries to prioritize public health in their patent-related decisions. It underscores the need for a balanced approach to intellectual property rights, innovation, and affordability in the realm of drug development.
